# Expression of cancer–testis antigens in esophageal cancer and their progress in immunotherapy

**DOI:** 10.1007/s00432-019-02840-3

**Published:** 2019-01-17

**Authors:** Yujie Zhang, Yuxin Zhang, Li Zhang

**Affiliations:** 1 Department of Oncology, Tongji Medical College, Tongji Hospital, Huazhong University of Science and Technology, No. 1095 Jiefang Avenue, Wuhan, 430030 Hubei China; 2 Hepatic Surgery Center, Tongji Medical College, Tongji Hospital, Huazhong University of Science and Technology, No. 1095 Jiefang Avenue, Wuhan, 430030 Hubei China

**Keywords:** Cancer–testis antigens, Esophageal cancer, MAGE-A, NY-ESO-1, LAGE-1, TTK

## Abstract

**Purpose:**

Esophageal cancer is a common disease in China with low survival rate due to no obvious early symptoms and lack of effective screening strategies. Traditional treatments usually do not produce desirable results in patients with advanced esophageal cancer, so immunotherapy which relies on tumor-related antigens is needed to combat low survival rates effectively. Cancer–testis antigens (CTA), a large family of tumor-related antigens, have a strong in vivo immunogenicity and tumor-restricted expressing patterns in normal adult tissues. These two characteristics are ideal features of anticancer immunotherapy targets and, therefore, promoted the development of some studies of CTA-based therapy. To provide ideas for the role of the cancer–testis antigens MAGE-A, NY-ESO-1, LAGE-1, and TTK in esophageal cancer, we summarized their expression, prognostic value, and development in immunotherapy.

**Methods:**

The relevant literature from PubMed is reviewed in this study.

**Results:**

In esophageal cancer, although the relationship between expression of MAGE-A, NY-ESO-1, LAGE-1, and TTK and prognosis value is still in a controversial situation, MAGE-A, NY-ESO-1, LAGE-1, and TTK are highly expressed and can induce specific CTL cells to produce particular killing effect on tumor cells, and some clinical trials have demonstrated that immunotherapy for esophageal cancer patients is effective and safe, which provides a new therapeutic strategy for the treatment of esophageal cancer in the future.

**Conclusion:**

In this review, we summarize expression and prognostic value of MAGE-A, NY-ESO-1, LAGE-1, and TTK in esophageal cancer and point out recent advances in immunotherapy about them.

## Introduction

Esophageal cancer was one of the most common cancers in China and became one of the major health threats. Owing to the deficiency of effective screening strategies and lack of symptoms in early-stage of esophageal cancer, patients were often diagnosed at an advanced stage, and it was ranked fourth in the five leading causes of cancer-related deaths in China (Chen et al. 2017). Currently, treatment options for patients with esophageal cancer are limited to three basic modalities: surgical resection, radiotherapy, and chemotherapy (Enzinger and Mayer 2003), which usually do not produce desirable results in patients with advanced cancer (Suntharalingam 2006). Therefore, new therapeutic approaches are needed to combat low survival rates effectively.

One of the promising strategies is immunotherapy, which relies on tumor-related antigens. Immunotherapy against tumor-associated antigens, using the body’s immune cells to recognize tumor-related antigens, has become one of the most desirable treatment options for esophageal cancer patients. Furthermore, tumor immunotherapy mainly depends on the amount of antigens expression and the response of T cells to tumor antigens, so these two characteristics of the tumor antigens become the basis of tumor immunotherapy. Among all tumor-associated antigens, cancer–testis antigens such as MAGE-A, NY-ESO-1, LAGE-1, and TTK are of particular concern as a potential target for immunotherapy by reason of their strong in vivo immunogenicity and unique expression patterns. Cancer–testis (CT) antigens are usually expressed only in the testis, except for expression in early-developing embryos and placentas. In addition, CT antigens are also expressed in various tumor types (Whitehurst 2014). Since the testis is an immunoprivileged site for low expression of HLA molecules, CT antigens are the ideal targets for tumor immunotherapy, including cancer vaccination, adoptive T-cell transfer with chimeric T-cell receptors and immuno-check point inhibitors (Gordeeva 2018). The development of cancer-specific immunotherapy has been ongoing for many years, and several candidate targets such as MAGE-A, NY-ESO-1, LAGE-1, and TTK have entered early clinical trials. In patients with improved survival outcomes, T cells and antibodies responsive to tumor CT antigens have been detected to support this anti-cancer immunotherapy.

Hence, this review aims to focus on CT antigens MAGE-A, NY-ESO-1, LAGE-1, and TTK, and discuss their expression, prognostic value and targeted immunotherapeutic strategies in esophageal cancer.

### MAGE-A

The MAGE-A (Melanoma-associated antigens-A) subfamily is a member of the MAGE gene family and belongs to cancer–testis antigens, including MAGE-A1–MAGE-A12 (Meek and Marcar 2012), which is localized on the X-chromosome. Notably, proteins of the MAGE family were the first recognized members of CT antigens. The antigenic peptide encoded by the MAGE-A gene can be presented to cytotoxic T cells by MHC I molecules of cancer cells and can exert specific anti-tumor activity (Schooten et al. 2018). MAGE-A are almost not expressed in normal tissues, but expressed in various tumor tissues (Schultz-Thater et al. 2011; Mengus et al. 2013; Ayyoub et al. 2014), which have high expression in esophageal carcinoma cell lines and esophageal carcinoma tissues.

Therefore, with MAGE-A antigen as a new target, it is a good prospect for immunotherapy of patients with esophageal cancer.

## The expression of MAGE-A in esophageal cancer

### Gene level

In the study of Liang Zhen’s team, 11 kinds of cancer–testis antigen genes were analyzed, and no antigen was expressed in the normal esophageal mucosa. However, in esophageal cancer tissues, MAGE-3 expression was the most frequent (62.9%), followed by MAGE-4 (31.4%), MAGE-1 (25.7%). As tumors progressed, the expression rates of MAGE-1, MAGE-3, and MAGE-4 increased (Liang et al. 2005). The results of another team showed that MAGE-A4 mRNA was overexpressed in 90.2% of esophageal cancer specimens. The above results indicate that the gene expression rate of MAGE-A subfamily is relatively high in esophageal cancer tissues (Forghanifard et al. 2011).

### Protein level

The Argun Akcakanat team used immunohistochemistry to analyze the expression of MAGE protein in tissue samples from 213 patients with esophageal cancer. The results showed that anti-MAGE antibody staining positive was observed in 111 specimens (52%). What’s more, the immune reactivity of anti-MAGE antibody was strong, and 73 (66%) of the MAGE-A positive sections showed tumor cell staining > 50% (Akcakanat et al. 2006). In another study, immunohistochemical analysis of MAGE-A protein expression in 98 patients with esophageal cancer showed that positive reaction in 5 out of 32 adenocarcinomas (15%) and 33 out of 66 (50%) esophageal squamous cell carcinoma (ESCC) (Bujas et al. 2011). In a previous study, IHC staining showed 50% MAGE-A protein expression in esophageal cancer (Haier et al. 2006), similar to 58% of the findings in Chen et al. (2014) study.

### Tissue level

Bujas et al. selected 55 patients with ESCC and 28 (50.9%) patients had 1 or more lymph node metastases. Immunohistochemical expression of primary tumor and metastatic lymph node specimens showed that MAGE-A 3/4 was expressed in 90.9% of primary ESCC and all metastatic lymph nodes. High expression in lymph node metastasis suggests that post-operative vaccines using MAGE-A in the advanced stages of the disease may have clinical benefit (Bujas et al. 2011). In another study, 38.8% of the 98 patients showed MAGE-A expression. 87% of them were squamous cell carcinoma, and 13.2% (5/38) were adenocarcinoma (Haier et al. 2006). Similarly, Kerkar et al. have come to the same conclusion (Kerkar et al. 2016). Since MAGE-A is an X-chromosome-associated antigen, the researchers compared its expression in both female and male tumor tissues. The results suggested that the positive rate of MAGE-A in the two sexes was similar (Haier et al. 2006). Sang et al. found that 59.3% of esophageal squamous cell carcinoma specimens showed MAGE-A11 expression, and the expression of MAGE-A11 was positively correlated with distant lymph node metastasis. The overall survival rate of MAGE-A-11-positive ESCC patients was shorter than that of MAGE-A-11-negative patients (Sang et al. 2016).

In general, the expression of MAGE-A has tumor specificity and it is comparatively higher in ESCC than esophageal adenocarcinoma. Due to the largest number of esophageal cancer is ESCC in China, researchers can make full use of the high expression characteristics of MAGE-A gene in esophageal squamous cell carcinoma for specific immunotherapy. Therefore, MAGE-A may represent the potential immunotherapy target of esophageal cancer.

## The relationship between MAGE-A expression and prognosis of esophageal cancer

Akcakanat et al. illustrate that the lack of MAGE-A expression has a prognostic value in estimating the overall survival rate of all patients. Patients with MAGE-A negative tumors had a slightly better prognosis. However, the prognosis difference between MAGE-A positive tumor and MAGE-A negative tumor was not significant (*p* = 0.1756). The 3-year survival rates of MAGE-A antigen positive and negative patients were 46.1% and 57.6%, respectively. There was no correlation between cancer testicular antigen expression and disease progression or TNM factors (Akcakanat et al. 2006). Similarly, Haier et al. also did not find an association between MAGE-A expression score and tumor differentiation, TNM or UICC staging (Haier et al. 2006).

However, Forghanifard et al. showed that the expression level of MAGE-A4 is related to the parameters of tumor progression. Its expression level is not only associated with tumor metastasis to lymph nodes, but also the number of metastatic lymph nodes. Up-regulation of MAGE-A4 may enhance the ability of tumor cells to invade and metastasize (Forghanifard et al. 2011). In addition, Sang et al. utilize multivariate Cox regression analysis to show that MAGE-A11 expression is an independent adverse prognostic factor in patients with esophageal squamous cell carcinoma. Overexpression of MAGE-A11 alters multiple gene expression, which is involved in a variety of cellular functions such as protein ubiquitination, cell proliferation, and apoptosis, tumor invasion and metastasis. Overexpression of MAGE-A11 increases the ability of invasion and proliferation of esophageal squamous carcinoma cells (Sang et al. 2016).

Overall, the relationship between MAGE-A expression and prognosis of esophageal cancer is still in a controversial situation.

## The progress of MAGE-A molecule in immunotherapy of esophageal cancer

Due to the expression characteristics of MAGE-A in tissues and its high expression level in esophageal cancer, the researchers have made great efforts to develop cancer vaccines and adoptive T-cell transfer towards MAGE-A in esophageal cancer.

### Therapeutic cancer vaccines

One way to fight cancer using the patient’s immune system is to take advantage of cancer therapeutic vaccines. Cancer therapeutic vaccines are valid immunotherapy designed to delay or lessen tumor growth. Many types of cancer therapeutic vaccines have been used over the years, including protein or peptide vaccines, cell-based vaccines, DNA or RNA vaccines, and vector-based vaccines (Schooten et al. 2018). A preclinical study showed that among 15 genetically diverse and outbred mice immunized with the MAGE-A vaccine, 14 of them induced immune responses and produced a cross-reactive immune response. The MAGE-A DNA therapeutic vaccine remarkably slowed down the growth of the tumor and doubled the median survival rate of mice. These findings support the clinical application of consensus MAGE-A immunogens targeting multiple MAGE-A family members to avoid tumor immune-escape (Duperret et al. 2018). Since dendritic cells (DCs) are the primary antigen-presenting cells and are strong activators of T cells, many studies have studied the use of peptide-pulsed DC as cell vaccines. Forghanifard et al. (2014) in vitro transferred the cancer–testis antigen mRNA to the ex vivo dendritic cells from the patients of esophageal squamous cell carcinoma, and the cytotoxic T-lymphocytes of the patients can be activated by dendritic cells, which caused T-cell anti-tumor cytotoxic effects.

Clinical trials of MAGE-A vaccines have been conducted in melanoma (van Baren et al. 2005; Bonehill et al. 2009; Wilgenhof et al. 2011; Kruit et al. 2013; Kranz et al. 2016; Grob et al. 2017), lung cancer (Tyagi and Mirakhur 2009; Vansteenkiste et al. 2013, 2016; Pujol et al. 2015), colon cancer (Takahashi et al. 2012) and myeloma (Rapoport et al. 2014), and anti-tumor effects have been observed in some of these clinical trials. However, at present, clinical trials of MAGE-A vaccines have not been reported in esophageal cancer, which will be a promising treatment method and deserve the attention of researchers.

### Adoptive T-cell transfer (ACT)

A preclinical research showed that TCR therapy with HLA-A24 restrictive TCR against MAGEA4 143–151 peptide (NYKRCFPVI) is a prospective strategy for the treatment of MAGE-A4-expressing tumors. This study demonstrated that genetically engineered T cells expressing MAGE-A4-specific TCR could prevent the growth of esophageal cancer expressed MAGE-A4 in immunodeficient NOG mice (Shirakura et al. 2012). In a phase I dose-escalating study, after the Shinichi Kageyama team transferred the TCR gene into T cells, the T cells were returned to patients with recurrent esophageal cancer expressing MAGE-A4, and then the patients were given a sequential MAGE-A4 peptide vaccine. This scheme does not include lymphocyte depletion and the use of IL-2. In adoptive T-cell therapy, lymphocyte depletion is used to reduce the number of immunosuppressive cells and to lessen competition for activating cytokines. The results showed that seven patients presented tumor progression within 2 months after treatment and three patients with minimal tumor lesions at baseline survived for more than 27 months (UMIN000002395) (Kageyama et al. 2015). Lu et al. evaluated the safety and effectiveness of adoptive CD4+ T-cell therapy, which used the MHC-II restriction and HLA-DPB1*0401-restricted TCR to identify MAGE-A3. The objective partial response of 4 months was observed in a patient with esophageal cancer treated with the highest dose of TCR-transduced CD4+ T cells (Lu et al. 2017). A clinical trial in which nine cancer patients including one with esophageal carcinoma were treated with autologous TCR-engineered T cells targeting the HLA-A2 restricted CT antigen MAGE-A3 (amino acid 112–120; KVAELVHFL) indicated encouraging results (NCT01273181) (Morgan et al. 2013).

Together with the results of ongoing trials, this will certainly further improve clinical efficacy and safety of MAGE-directed treatment in the near future. Although clinical trials against MAGE-A antigens have so far only shown clinical responses in subgroups of patients, preclinical data shows great prospect of immunotherapy against tumors that express MAGE. As great advances have been made in understanding the underlying mechanisms of tumor immune-escape, as well as lessons learned from safety-related clinical research, this has created a time when cancer immunotherapy can make significant improvements in efficacy and safety.

#### NY-ESO-1

The gene encoding New York’s esophageal squamous cell carcinoma 1 (NY-ESO-1), also known as cancer/testis antigen 1B (CTAG1), is a prototype of the cancer–testis (CT) gene family that was initially separated from esophageal cancer. The main characteristics of NY-ESO-1 are not expressed in the normal tissues, only in or selectively expressed in the testis and many malignancies (Jungbluth et al. 2001), including melanoma (Prasad et al. 2004; Aung et al. 2014), lung (Gure et al. 2005; Grunwald et al. 2006; Chapman et al. 2011; Shan et al. 2013), ovary (Odunsi et al. 2003) and so on. Besides, the positive rate of s-NY-ESO-1-Abs in esophageal cancer patients was significantly higher than that of other types of cancer (Oshima et al. 2016). This expression pattern makes it a suitable target for cancer immunotherapy.

## The expression of NY-ESO-1 in esophageal cancer

### Gene level

In a study involving 123 ESCCs, NY-ESO-1 mRNA was expressed in 41 (33%) cancer specimens. No expression of NY-ESO-1 mRNA was observed in 123 adjacent normal esophageal tissues. The expression frequency of NY-ESO-1 mRNA was significantly higher in well-differentiated and moderately differentiated esophageal cancer than in poorly differentiated types. Higher frequency of NY-ESO-1 mRNA expression was observed in the late stage of cancer. However, the difference between I/II and III/IV was not statistically significant (Fujita et al. 2004). In another study, the researchers extracted RNA from fresh esophageal cancer tissue before other therapeutic interventions in 41 patients with esophageal squamous cell carcinoma (ESCC). The expression of NY-ESO-1 mRNA was found in 41.4% ESCC specimens (Forghanifard et al. 2011).

### Protein level

Akcakanat et al. detected the expression of CTA protein in 213 patients with esophageal cancer by immunohistochemistry. NY-ESO-1 was expressed in 44 patients (21%) (Akcakanat et al. 2006). Another team used the same method to analyze the expression of the NY-ESO-1 protein in 56 samples of esophageal cancer, and NY-ESO-1 protein expression was found in 18 patients (32%) (Akcakanat et al. 2004). Fujita et al. (2004) examined NY-ESO-1 protein expression by immunohistochemical staining in 64 cases of esophageal cancer, and 26 cases were positive (41%). Most NY-ESO-1 protein-positive cancer specimens were classified into highly differentiated and moderately differentiated tissues by histopathological examination and classified into stage III and IV. Another study included 55 patients with ESCC who underwent radical surgery and immunohistochemistry was used to detect the NY-ESO-1 protein expression in primary tumor and metastatic lymph node specimens. The result showed that 53 cases (96.6%) of the primary tumor were NY-ESO-1 positive and two examples (7.1%) of the metastatic lymph node were negative for NY-ESO-1. In the primary tumor and metastatic lymph nodes, the immunohistochemical expression of NY-ESO-1 is restricted to tumor cells and cytoplasm. This study showed that the expression of NY-ESO-1 in primary tumors was positively correlated with the expression of NY-ESO-1 in metastatic lymph nodes (*p* = 0.001), and negatively correlated with the age of patients (*p* < 0.001) (Bujas et al. 2011).

### Tissue level

In 27 cases of esophageal adenocarcinoma resection, 4 cases (15%) showed diffuse cytoplasmic and nuclear expression of NY-ESO-1 (Hayes et al. 2014). In another study, immunohistochemical staining was performed on paraffin-embedded tissues of 61 esophageal cancers, including 40 adenocarcinomas and 21 squamous carcinomas. The positive staining for adenocarcinoma and squamous cell carcinoma was 10% and 19%, respectively (Chen et al. 2014).

## The relationship between NY-ESO-1 expression and prognosis of esophageal cancer

Survival data from Fujita et al. (2004) showed that the survival rate of NY-ESO-1 protein-positive cases was higher than negative cases, but the difference was not statistically significant. The data from Akcakanat et al. (2004) showed that the 3-year survival rates of NY-ESO-1 antibody-positive and antibody-negative patients were 50% and 61%, respectively, and there was no statistically significant difference in survival between the two groups. Similarly, there was no notable association between the expression of NY-ESO-1 in esophageal squamous cell carcinoma and the prognostic parameters such as TNM stage, survival rate (Bujas et al. 2011) and clinicopathological features (Forghanifard et al. 2011). The high expression levels of MAGE-A3 and NY-ESO-1 in metastatic lymph nodes suggest that post-operative vaccine may have clinical benefit in advanced tumors. In addition, the expression of NY-ESO-1 in tumors was negatively correlated with the age of the patient, indicating a better response to treatment in young patients (Bujas et al. 2011).

In conclusion, NY-ESO-1 may be associated with the patients’ prognosis, but the difference is not statistically significant. Thus, the relationship between the expression of NY-ESO-1 and the prognosis of esophageal cancer patients is still disputable.

## The progress of NY-ESO-1 molecule in immunotherapy of esophageal cancer

NY-ESO-1 is restricted in normal tissues and is relatively high in tumor tissues, making it a target with limited off-target toxicity and a good candidate for immunotherapy. NY-ESO-1 has achieved some promising results in early I/II studies and has significant application value in esophageal cancer.

### Therapeutic cancer vaccines

In a study by Forghanifard et al., a chimeric sequence was synthesized using cytotoxic HLA-restricted epitopes of MAGE-A4, NY-ESO-1, and LAGE-1 as a target. The chimeric mRNA pool was transcribed in vitro and electroporated into monocyte-derived DCs. In three ESCC patients who had not undergone any treatment intervention before surgery, the cytotoxicity of CTLs induced by DCs carrying chimeric mRNA was significantly higher than that of mock DCs (*p* < 0.05) (Forghanifard et al. 2014). In the study of Kageyama et al., 25 patients participated in clinical trials, all of whom had unresectable, advanced or refractory esophageal cancer. All the tumor cells in these patients were NY-ESO-1 positive. Cholesterol amylopectin (CHP) is a novel antigen delivery system for cancer vaccines. Thirteen and twelve patients were repeatedly inoculated with 100 µg or 200 µg of NY-ESO-1 protein combined with CHP. The dose of 200 µg was more effective in inducing an immune response and suggesting better survival benefits. The progression-free survival time of the disease averaged 11 weeks (Kageyama et al. 2013). The results of Uenaka et al. showed that inoculation of four esophageal cancer patients with a complex of cholesterol-containing hydrophobic amylopectin and NY-ESO-1 protein (CHP-NY-ESO-1) caused antibody responses. An anti-tumor response was observed in three esophageal cancer patients. The use of whole proteins containing multiple CD4 and CD8 epitopes may be beneficial for cancer vaccines to prevent tumors from escaping immune responses (Uenaka et al. 2007). In another study, a phase I clinical trial of the NY-ESO-1f peptide vaccine included six patients with esophageal cancer to assess vaccine safety, immune response and tumor response, one of which was in a stable condition. Studies have shown that the NY-ESO-1f peptide vaccine was well tolerated and induces humoral, CD4 and CD8 T-cell responses in immunized patients (Kakimi et al. 2011).

### Adoptive T-cell transfer

A phase II, open-label, ATTACK-OG (NCT01795976) trial is using ACT assessment of autologous T cells from patients with esophageal cancer expressing NY-ESO-1, which is genetically modified to target NY-ESO-1. In this trial, patients received pretreatment chemotherapy with cyclophosphamide followed by fludarabine, and the prepared NY-ESO-1 gene-modified T cells were then transfected into the same patient. Another phase I study (NCT02869217) also began to assess the safety and efficacy of TBI-1301 (NY-ESO-1 specific TCR gene-transduced autologous T cells) in patients with solid tumors that expressed NY-ESO-1, including EC (Tanaka et al. 2017; Thomas et al. 2018).

Since its discovery, researchers have explored NY-ESO-1 as an anti-cancer target for immune-based treatments. Some methods have been studied in vitro, in vivo, and clinical trials. Most clinical trials concentrate on advanced solid cancers. NY-ESO-1 targeted therapy has made considerable progress, using a variety of methods from peptide and protein vaccines to adoptive T-cell therapy and combined therapy mode to target antigens. Encouraging results have been achieved, promoting new clinical trials for esophageal cancer.

#### LAGE-1

The LAGE-1 gene is 3245 bp long and encodes three exons. LAGE-1a, 1b, 1L, and 1S are collectively referred to as LAGE-1 family genes (Lethe et al. 1998). The gene LAGE-1 is located in Xq28 and is situated near the MAGE-A1 gene of the MAGE family. Research findings suggest that the expression of LAGE-1 is strongly correlated with the expression of NY-ESO-1 and MAGE genes. In addition, there is a common HLA-A*0201 epitope, 157–165, with NY-ESO-1[34]. LAGE-1 is also a cancer–testis antigen that is ideal for tumor immunotherapy because it is up-regulated in many tumor types including melanomas (Vaughan et al. 2004; Bolli et al. 2005; Kudela et al. 2011), ovary (Piura and Piura 2009; McCormack et al. 2013), lung (Bolli et al. 2005; Gure et al. 2005; Grunwald et al. 2006; McCormack et al. 2013), head and neck (Cuffel et al. 2011), prostate (Fossa et al. 2004; Hudolin et al. 2006) and bladder cancers (Sharma et al. 2006; Dyrskj et al. 2012) and so on, and highly restricted in normal tissues. Besides, LAGE-1 has a tumor-specific expression similar to that of MAGE-A1 and NY-ESO-1, so it is possible to become a target antigen for anti-tumor immunotherapy of esophageal carcinoma (Lethe et al. 1998).

## The expression of LAGE-1 in esophageal cancer

### Gene level

In a study, 39% of ESCC tumor specimens detected LAGE1 mRNA expression (Forghanifard et al. 2011). Similarly, Mashino et al. (2001) tested the expression of LAGE-1 gene in 46 esophageal cancer specimens, and the researchers found that the expression of LAGE-1 was relatively high (39.1%). In another study, it was found that 11 CT antigen genes were not expressed in the normal esophageal mucosa, and the LAGE-1 gene was expressed in esophageal carcinoma tissues (28.6%) (Liang et al. 2005).

### Tissue level

LAGE-1 is not expressed in normal tissues, but is found in 3/11 (27%) of esophageal cancer cell lines and 3/7 (43%) of esophageal cancer tissues, and is not shown in the normal esophageal epithelial tissue (Kan et al. 2006).

## The relationship between LAGE-1 expression and prognosis of esophageal cancer

The expression of LAGE-1 gene was not correlated with clinical-pathological parameters. In addition, there was no significant relationship between LAGE-1 expression and clinical parameters in esophageal cancer tissues (Mashino et al. 2001).

## The progress of LAGE-1 molecule in immunotherapy

Immunotherapy for LAGE-1 have been reported in other types of tumors, such as preclinical studies (McCormack et al. 2013) as well as adoptive cell transfer for melanoma (Kudela et al. 2011) and multiple myeloma (Rapoport et al. 2015), but there is no relevant study in esophageal cancer.

### TTK

TTK protein kinase (TTK), also called Monopolar spindle 1 kinase (MPS1), is a HLA-A2402-restricted epitope peptide derived from cancer–testis antigens (CTA) (Mizukami et al. 2008), and it is a previously unidentified member of the kinase family. It can phosphorylate serine, threonine, and tyrosine hydroxyamino acids, and is associated with cell proliferation (Tao et al. 2012) and cell invasion (King et al. 2018). It is also a critical regulator of the spindle assembly checkpoint (SAC), which functions to maintain genomic integrity (Thu et al. 2018) and controls cell fate (Szymiczek et al. 2017). TTK is expressed at relatively high levels in testis, thymus tissues and various malignant tumor tissues (Kaistha et al. 2014; Miao et al. 2016; Choi et al. 2017; Szymiczek et al. 2017; Chen et al. 2018; King et al. 2018), but is not detected in most other benign tissues (Mills et al. 1992). It is up-regulated in ESCC, which may be participated in tumor proliferation, progression and/or represent the specificity of ESCC. In many studies of ESCC antigens expression, the expression of TTK was observed as a key indicator for observing the clinical response of patients to cancer vaccination at present, and also as TTK inhibitors for ESCC patients in the future.

## The expression of TTK in esophageal cancer

### Gene level

The gene expression profile data of Kono et al. (2009) showed that TTK was overexpressed in a large portion (> 90%) of esophageal cancers, while it was almost undetectable in normal organs, except the testis and/or placenta. Similarly, two other studies have shown that TTK mRNA level is increased in esophageal cancer (Tao et al. 2012; He et al. 2018).

### Tissue level

Consistent with the results of gene expression, TTK was overexpressed in the great majority of ESCC tissues, but scarcely expressed in normal tissues (Yamabuki et al. 2006). Likewise, Mizukami et al. (2008) confirmed that the expression of TTK was frequently observed in ESCC tissues (100% for TTK), while no staining was observed in adjacent esophageal tissues.

## The relationship between TTK expression and prognosis of esophageal cancer

Using publicly available gene expression data, Tannous et al. (2013) determined that TTK overexpression corresponds positively with tumor grade and negatively with patient survival (two-sided *t* test, *p* < 0.001). He et al. (2018) indicate that this gene is vital in the progression of ESCC, but also decide the prognosis of patients. In other cancer types, the overexpression of the Mps1-encoding TTK gene was correlated with poor patients’ outcome of HCC (Choi et al. 2017), malignant mesothelioma (Szymiczek et al. 2017) and so on.

## The progress of TTK in immunotherapy of esophageal cancer

TTK used in the clinical study is considered to be very appropriate because it was expressed in the great majority (> 95%) of esophageal cancers, was expressed specifically in cancer cells and testis (cancer–testis antigens), was shown to be essential for the survival of cancer cells (Mizukami et al. 2008), and most importantly revealed very strong immunogenicity (Suda et al. 2007; Kono et al. 2009, 2012). Besides, it is also a vital regulator of the spindle assembly checkpoint (SAC), which functions to maintain genomic integrity (Thu et al. 2018) and controls cell fate (Szymiczek et al. 2017). These evidences strongly encouraged researchers to apply this CTA peptide as a candidate target for anti-cancer therapy.

### Therapeutic cancer vaccines

Iinuma et al. carried out phase I clinical study of multiple epitope peptide vaccines combined with chemoradiation therapy in 11 unresectable ESCC patients with HLA-A*2402. They selected five peptide vaccines including TTK to conquer the immune-escape mechanisms and improve the therapeutic potential. Researchers observed six patients of complete response (CR) and five patients of progressive disease (PD) after the eighth vaccination. The four CR cases who continued the vaccination experienced long consistent CR for more than 2 years.(Iinuma et al. 2014)Another phase I trial for nine patients with advanced ESCC was carried out for patients with HLA-A*2402 using epitope peptides derived from novel cancer–testis antigens, LY6K and TTK, in combination with CpG-7909. There were no complete response (CR) and partial response (PR). However, five patients showed stable disease (SD) (Iwahashi et al. 2010). Similarly, Kono et al. reported a phase I clinical cancer vaccination research in 10 HLA-A*2402 (+) patients with advanced ESCC who had been intractable to standard ESCC therapy, which combines multiple peptides that were derived from TTK, LY6K, and IMP-3. The median survival time after the vaccination was 6.6 months. Of the ten patients, 50% had a good clinical response after vaccination. One patient with hepatic metastasis experienced a CR lasting 7 months, one showed objective responses in all lung metastasis lesions, and three cases revealed a stable disease condition for at least 2.5 months (Kono et al. 2009). Next, Kono et al. conducted multicenter, phase II clinical trial of cancer vaccination for advanced esophageal cancer with three same peptides TTK, LY6K, and IMP-3. Sixty ESCC patients were enrolled to assess overall survival (OS), progression-free survival (PFS) and immunological response. The patients emerging the CTL induction for multiple peptides have good clinical responses (Kono et al. 2012).

The cancer vaccine therapy using TTK demonstrated satisfactory safety and good immunogenicity as well as promising disease control rate.

### TTK inhibitors

Lossing control of cell-cycle is a symbol of human cancer. Cell-cycle checkpoints are necessary for maintaining genome integrality and balanced growth and division. They are specifically deregulated in cancer cells and contain regulators that represent possible therapeutic targets. TTK, a genome-surveillance mechanism that is important for cell survival, and has emerged as a candidate target for anticancer therapy (Mason et al. 2017). Inhibition of TTK has emerged as a promising therapeutic strategy for the treatment of aneuploid tumors, with triple-negative breast cancer (TNBC) (Maia et al. 2015; Riggs et al. 2017; Thu et al. 2018; Zhu et al. 2018) and malignant mesothelioma (Szymiczek et al. 2017) important focus of clinical development. Human cancer cells treated with Mps1 inhibitor exhibit effects consistent with Mps1 kinase inhibition, specifically spindle assembly checkpoint inactivation, leading to chromosome missegregation, aneuploidy, and ultimately cell death (Mason et al. 2017).

Although TTK inhibitor is a promising treatment method, there are no published studies on the application of this therapy to esophageal cancer presently. We expect that researchers will focus this field on esophageal cancer in the future.

## Discussion

Esophageal cancer is frequently diagnosed at an advanced stage, so routine therapies cannot guarantee a satisfactory outcome as a result of relatively high postoperative recurrence and metastasis rate. How to control the growth of tumor cells and remove the residual tumor cells after therapy is an important clinical research direction for improving the survival rate of patients with esophageal cancer. Consequently, a promising therapeutic method, immunotherapy, is needed to deal with this problem. In esophageal cancer, MAGE-A, NY-ESO-1, LAGE-1, and TTK are highly expressed, and they can induce specific CTL cells to produce specific killing effect on tumor cells, and some clinical trials have demonstrated that tumor immunotherapy for esophageal cancer patients is effective, which provides a new therapeutic strategy for the treatment of esophageal cancer in the future.

Although immunotherapy is a good choice for patients with esophageal cancer, there are still some problems for individual patients. First, MAGE-A, NY-ESO-1, LAGE-1, and TTK are not expressed in all esophageal cancer patients as described above; second, whether the expression of these antigens in esophageal cancer patients can trigger an effective immune response to eliminate tumor cells; third, whether there are more effective peptides for MAGE-A, NY-ESO-1, LAGE-1 and TTK that can stimulate sufficient immune responses. To solve the above problems, future research could take the expression of other members of the cancer–testis antigens family in esophageal cancer into account, considering whether there is a correlation expression among each antigen to cover more esophageal cancer patients; whether epigenetic modifying agents can effectively improve the expression of cancer–testis antigens in esophageal cancer (James et al. 2006; Fratta et al. 2011; Rao et al. 2011; Kim et al. 2013; Linnekamp et al. 2017); using the development of computer bioinformatics technology to design the optimal peptides of MAGE-A, NY-ESO-1, LAGE-1 and TTK may significantly enhance the anti-tumor ability of CTL cells and produce ideal immune effect (Zhang et al. 2010).

The clinicopathological significance of cancer–testis antigens, especially MAGE-A, NY-ESO-1, LAGE-1, and TTK has gradually become clear through the efforts of many researchers, but the role of cancer–testis antigens in the development of esophageal cancer is still unclear. Explaining the interactions between these antigens, biological functions, and their mechanisms will lay a solid foundation for immunotherapy of esophageal cancer with the target of cancer–testis antigens. Currently, many research teams have conducted a number of clinical trials as mentioned above. Although the results are incomplete, immunotherapy will become one of the new treatment strategies for esophageal cancer in the future.
